# A phase I study of combined trabectedin and pegylated liposomal doxorubicin therapy for advanced relapsed ovarian cancer

**DOI:** 10.1007/s10147-021-01973-1

**Published:** 2021-06-30

**Authors:** Shunji Takahashi, Munetaka Takekuma, Kenji Tamura, Kazuhiro Takehara, Hiroyuki Nomura, Makiko Ono, Mayu Yunokawa, Daisuke Aoki

**Affiliations:** 1 Cancer Institute Hospital of the Japanese Foundation for Cancer Research, 3-8-31, Ariake, Koto-ku, Tokyo, 135-0063 Japan; 2 Shizuoka Cancer Center, 1007 Shimonagakubo, Nagaizumi-cho, Sunto-gun, Shizuoka, 411-8777 Japan; 3 National Cancer Center Hospital, 5-1-1 Tsukiji, Chuo-ku, Tokyo, 104-0045 Japan; 4 National Hospital Organization Shikoku Cancer Center, 160 Kou, Minamiumemotomachi, Matsuyama-shi, Ehime, 791-0245 Japan; 5 Keio University School of Medicine, 35 Shinanomachi, Shinjuku-ku, Tokyo, 160-8582 Japan; 6 Present Address: Shimane University Hospital, 89-1 Enya-cho, Izumo-shi, Shimane, 693-8501 Japan; 7 Present Address: Fujita Health University, 1-98 Dengakugakubo, Kutsukake-cho, Toyoake, Aichi 470-1192 Japan

**Keywords:** Trabectedin, Liposomal doxorubicin, Ovarian cancer, Recurrence, Phase I, Japan

## Abstract

**Background:**

Advanced relapsed ovarian cancer has a poor prognosis, and treatment options are limited.

**Methods:**

This phase I trial investigated the dosage, safety, pharmacokinetics and efficacy of trabectedin plus pegylated liposomal doxorubicin (PLD) in Japanese patients with advanced relapsed ovarian, fallopian tube, or primary peritoneal cancer. Patients received trabectedin 0.9 or 1.1 mg/m^2^ immediately after PLD 30 mg/m^2^; both drugs were given by intravenous infusion. Treatment was repeated every 21 days until disease progression or unacceptable toxicity. The maximum tolerated dose (MTD) was determined in an initial dose escalation phase, and this was used in a subsequent safety assessment phase. Safety and tumor response were monitored throughout the trial, and drug concentrations for pharmacokinetic analysis were measured during cycle 1.

**Results:**

Eighteen patients were included. The MTD of trabectedin was determined as 1.1 mg/m^2^. Gastrointestinal adverse events were experienced by all patients, but were mostly grade 1 or 2 in intensity. Most patients had grade ≥ 3 elevations in transaminase levels or grade ≥ 3 reductions in neutrophil count, but these events were generally manageable through dose reduction and/or supportive therapies, as appropriate. There were no deaths during the trial. Trabectedin exposure increased in a dose-dependent manner. The overall response rate was 27.8%.

**Conclusions:**

Trabectedin, in combination with PLD, may have clinical benefits in Japanese patients with relapsed advanced ovarian cancer. The recommended dosage of trabectedin for further study in this population is 1.1 mg/m^2^ once every 21 days.

*Clinical trial registration number*: JapicCTI-163164

**Supplementary Information:**

The online version contains supplementary material available at 10.1007/s10147-021-01973-1.

## Introduction

Ovarian cancer (OC) is a major cause of cancer-related death among women [1], accounting for over 150,000 deaths worldwide in 2012. Prognosis remains poor, with 5-year survival rates of 30–40% [1].

Treatment of advanced OC consists of cytoreductive surgery with chemotherapy, typically paclitaxel plus carboplatin [2]. Although initial treatment is often effective, approximately 70% of women will experience relapse within 3 years [2].

Subsequent treatment is largely dictated by platinum-free interval (PFI) [2]. In women who relapse within 6 months, there is evidence to support (non-platinum) monotherapy, but response rates are poor. The standard of care when PFI is ≥ 6 months is platinum-based chemotherapy, but evidence of survival benefit has been found with other combinations such as trabectedin + pegylated liposomal doxorubicin (PLD) [2, 3].

Trabectedin binds to the minor groove of DNA, and its mechanisms of action include indirect anti-inflammatory and anti-angiogenic activity via tumor-associated macrophages [4]. It has been approved in the European Union for the treatment of relapsed platinum-sensitive OC, at a recommended dose (RD) of 1.1 mg/m^2^ every 21 days in combination with PLD [5]. To date, no formal studies of trabectedin in Japanese patients with OC have been undertaken.

We, therefore, conducted a phase I trial to determine the RD of trabectedin (in combination with PLD) in Japanese patients and to investigate its safety, tolerability, pharmacokinetics and efficacy in this population.

## Patients and methods

### Study design and participants

**Fig. 1:**
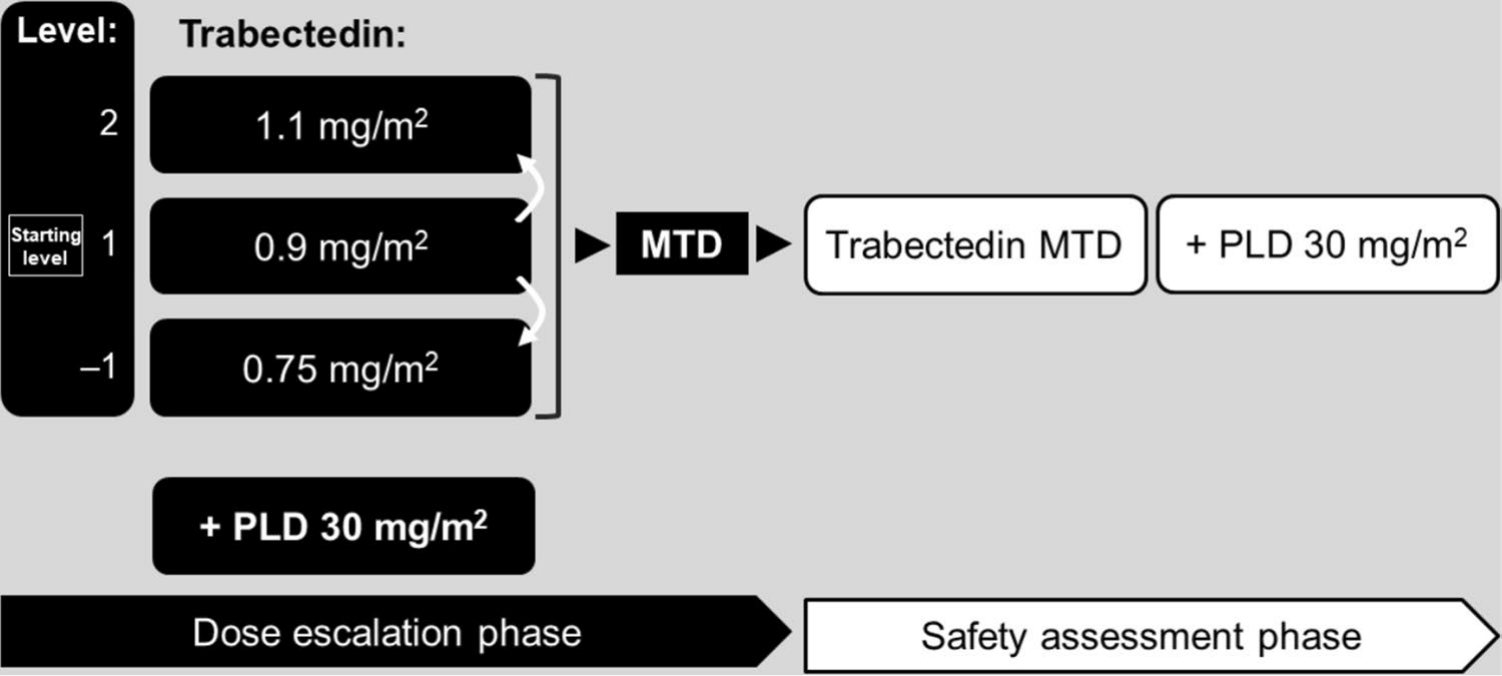
Study design. MTD was defined as the maximal dose level at which the incidence of dose-limiting toxicity did not exceed 33%. *MTD* maximum tolerated dose, *PLD* pegylated liposomal doxorubicin

This open-label, non-randomized study was conducted at five centers in Japan. It had two phases: (i) a dose escalation phase (DEP), to investigate the maximum tolerated dose (MTD) of trabectedin in combination with PLD; and (ii) a safety assessment phase (SAP) for further evaluation of safety at the MTD (Fig. 1). The trial was registered on www.clinicaltrials.jp (identifier JapicCTI-163164). All patients gave written informed consent to participate. The trial was conducted in accordance with the Declaration of Helsinki and was approved by the Institutional Review Board at each participating center.

Eligible patients were aged ≥ 20 years, had an Eastern Cooperative Oncology Group performance status of 0 or 1, and had a histologically confirmed diagnosis of epithelial ovarian, primary peritoneal, or fallopian tube cancer that had relapsed following treatment with platinum-based chemotherapy. Only patients not expected to benefit from re-treatment with platinum-based chemotherapy were included. Patients were excluded if they had received a cumulative dose of doxorubicin (or other anthracycline) > 300 mg/m^2^.

### Treatment and procedures

On Day 1 of each cycle, patients received premedication with intravenous (IV) dexamethasone and a 5-HT_3_ receptor antagonist; this was followed 30 min later by PLD, infused over 90 min. Trabectedin was then administered via a central venous line over 3 h, and follow-up continued for 20 days. Study drug administration was repeated every 21 days as long as clinical benefit was derived or until disease progression.

The starting dose of trabectedin in the DEP was 0.9 mg/m^2^ (level 1); the maximum dose was 1.1 mg/m^2^ (level 2), and the minimum dose (used when dose reduction was indicated; see below) was 0.75 mg/m^2^ (level –1; Fig. 1). The starting dose of PLD was 30 mg/m^2^ for all patients.

Recruitment of patients to level 2 occurred if the incidence of dose-limiting toxicities (DLTs; defined in Supplementary Table S1) at level 1 was not > 33%. Three patients were planned for enrolment at level 1, with three more being enrolled in the event of a DLT (i.e. a 3 + 3 design) [6]. At least six patients were treated at the presumed MTD of trabectedin, which was defined as the highest dose not associated with a DLT incidence > 33% [6]. For the purposes of MTD determination, DLTs were evaluated only during cycle 1 in the DEP.

Once the MTD had been determined, six additional patients received trabectedin (plus PLD) at the MTD in the SAP (Fig. 1). The RD of trabectedin was determined by evaluating the results from both phases of the trial.

Stepwise dosage reduction was triggered if any adverse event (AE) from a prespecified list occurred (Supplementary Table S2). Doses were not increased in individual patients.

### Outcomes

The primary endpoint was the incidence of DLT. Secondary endpoints were safety, pharmacokinetic parameters, and efficacy outcomes. AEs were graded according to the National Cancer Institute Common Terminology Criteria for Adverse Events (version 4.03) [7].

Blood samples for pharmacokinetic analysis were taken at predetermined intervals from Day 1 to Day 8 of cycle 1. The pharmacokinetic parameters calculated (using a non-compartmental model) were maximum concentration (*C*_max_), time to *C*_max_ (*t*_max_), area under the concentration–time curve from zero to infinity (AUC_0–inf_), terminal half-life (*t*_½_), clearance (CL), and volume of distribution at steady state (Vd_ss_).

Tumor response was assessed according to the Response Evaluation Criteria in Solid Tumors (RECIST) guidelines (version 1.1) [8] at screening, every 6 weeks until week 24, and every 9 weeks thereafter.

### Statistical analysis

Sample size was not based on statistical calculations, but was chosen to be sufficient to evaluate the tolerability of trabectedin plus PLD. We carried out primary analyses on the DLT-evaluable population (all patients who were enrolled in the DEP and received predefined doses of trabectedin or PLD). Safety analyses were done on all patients who received at least one dose of study drug (trabectedin or PLD), whereas efficacy analyses were done on the full analysis set (all treated patients with histologically confirmed cancer). All analyses were carried out using SAS v9.4 (SAS Institute, Inc., Cary, NC, USA).

## Results

### Patient characteristics

Eighteen patients were enrolled: 12 patients in the DEP (level 1: *n* = 6; level 2: *n* = 6) and six patients in the SAP (level 2). The progression of the trial is depicted in Supplementary Fig. S1.

All patients had undergone surgery and received ≥ 1 prior line of chemotherapy. Fifteen patients (4 in level 1, and 11 in level 2) had a PFI of < 6 months at the time of enrolment (Table 1).

**Table 1:** Patient characteristics

	Level 1 (*n* = 6)	Level 2 (*n* = 12)	Total (*n* = 18)
Age, years	63.0 (7.8)	50.3 (11.9)	54.5 (12.2)
Body weight, kg	49.8 (6.1)	58.1 (9.0)	55.4 (8.9)
ECOG performance status			
0	6 (100.0)	8 (66.7)	14 (77.8)
1	0	4 (33.3)	4 (22.2)
Primary tumor location and histology			
Ovary			
Clear cell carcinoma	1 (16.7)	2 (16.7)	3 (16.7)
Mixed epithelial tumor	0	1 (8.3)	1 (5.6)
Papillary/serous	1 (16.7)	9 (75.0)	10 (55.6)
Peritoneum	2 (33.3)	0	2 (11.1)
Fallopian tube	2 (33.3)	0	2 (11.1)
Histological grade			
Grade 3	1 (16.7)	3 (25.0)	4 (22.2)
Unknown	5 (83.3)	9 (75.0)	14 (77.8)
Stage			
III	5 (83.3)	6 (50.0)	11 (61.1)
IV	1 (16.7)	5 (41.7)	6 (33.3)
Unknown	0	1 (8.3)	1 (5.6)
Platinum-free interval, months			
< 6	4 (66.7)	11 (91.7)	15 (83.3)
6– < 12	2 (33.3)	1 (8.3)	3 (16.7)
Number of prior treatment regimens			
1	2 (33.3)	1 (8.3)	3 (16.7)
2	1 (16.7)	3 (25.0)	4 (22.2)
3	2 (33.3)	3 (25.0)	5 (27.8)
≥ 4	1 (16.7)	5 (41.7)	6 (33.3)
PLD as prior treatment	0	4 (33.3)	4 (22.2)

Categorical variables are expressed as *n* (%) and continuous variables as mean (SD)

*ECOG* Eastern Cooperative Oncology Group, *PLD* pegylated liposomal doxorubicin, *SD* standard deviation

Patients enrolled in levels 1 and 2 received 1–21 (median, 5.0) cycles and 1–14 (median, 5.0) cycles of treatment, respectively. The median relative dose intensity for trabectedin was 74.6% in level 1 and 78.2% in level 2; for PLD, it was 74.6% in level 1 and 80.1% in level 2. Trabectedin dosage was reduced in 6 patients (1 in level 1, and 5 in level 2); in all cases, this was in response to an AE. Reasons for study discontinuation were radiological disease progression (*n* = 9; 3 in level 1 and 6 in level 2), clinical disease progression (*n* = 3; all in level 2), AEs (*n* = 5; 3 in level 1 and 2 in level 2), and patient withdrawal of consent (*n* = 1; level 2).

### Safety

All 12 patients who participated in the DEP were evaluable for DLT. One patient in level 1 experienced a DLT, namely a grade 4 reduction in neutrophil count lasting more than 6 days. No patients in level 2 experienced a DLT. Therefore, the MTD of trabectedin was 1.1 mg/m^2^ (level 2); this dose was used for the SAP.

All patients reported AEs (Table 2). Individual AEs occurring in ≥ 50% of patients were anaemia (3/6 and 7/12 in levels 1 and 2, respectively), nausea (6/6 and 11/12), decreased appetite (6/6 and 7/12), vomiting (4/6 and 8/12), stomatitis (3/6 and 7/12), alanine aminotransferase (ALT) increased (4/6 and 12/12), aspartate aminotransferase (AST) increased (3/6 and 12/12), gamma-glutamyltransferase increased (2/6 and 7/12), neutrophil count decreased (6/6 and 11/12), platelet count decreased (2/6 and 7/12), and white blood cell count decreased (5/6 and 9/12). Grade 3 or 4 AEs occurring in ≥ 30% of patients were ALT increased (3/6 and 12/12 in levels 1 and 2, respectively), AST increased (2/6 and 12/12), neutrophil count decreased (6/6 and 9/12), and white blood cell count decreased (5/6 and 8/12). Three grade 3 or 4 gastrointestinal events were recorded (abdominal distension [level 2], rectal perforation [level 2], and stomatitis [level 1]).

**Table 2:** Adverse events occurring in ≥ 20% of study participants in any dose level

Adverse event	Level 1 (*n* = 6)		Level 2 (*n* = 12)		Total (*n* = 18)	
	All grades	Grade ≥ 3	All grades	Grade ≥ 3	All grades	Grade ≥ 3
Any adverse event	6 (100.0)	6 (100.0)	12 (100.0)	12 (100.0)	18 (100.0)	18 (100.0)
System organ class						
*Preferred term*						
Blood and lymphatic system disorders						
Anemia	3 (50.0)	1 (16.7)	7 (58.3)	4 (33.3)	10 (55.6)	5 (27.8)
Febrile neutropenia	0	0	3 (25.0)	3 (25.0)	3 (16.7)	3 (16.7)
Gastrointestinal and related disorders						
Constipation	4 (66.7)	0	3 (25.0)	0	7 (38.9)	0
Diarrhoea	1 (16.7)	0	3 (25.0)	0	4 (22.2)	0
Nausea	6 (100.0)	0	11 (91.7)	0	17 (94.4)	0
Stomatitis	3 (50.0)	1 (16.7)	7 (58.3)	0	10 (55.6)	1 (5.6)
Vomiting	4 (66.7)	0	8 (66.7)	0	12 (66.7)	0
General disorders and administration site conditions						
Fatigue	2 (33.3)	0	5 (41.7)	0	7 (38.9)	0
Malaise	2 (33.3)	0	5 (41.7)	0	7 (38.9)	0
Edema peripheral	1 (16.7)	0	3 (25.0)	0	4 (22.2)	0
Pyrexia	2 (33.3)	0	2 (16.7)	0	4 (22.2)	0
Infections and infestations						
Upper respiratory tract infection	3 (50.0)	0	3 (25.0)	0	6 (33.3)	0
Investigations						
ALT increased	4 (66.7)	3 (50.0)	12 (100.0)	12 (100.0)	16 (88.9)	15 (83.3)
AST increased	3 (50.0)	2 (33.3)	12 (100.0)	12 (100.0)	15 (83.3)	14 (77.8)
Blood CPK increased	0	0	6 (50.0)	2 (16.7)	6 (33.3)	2 (11.1)
Blood LDH increased	0	0	3 (25.0)	0	3 (16.7)	0
GGT increased	2 (33.3)	1 (16.7)	7 (58.3)	3 (25.0)	9 (50.0)	4 (22.2)
Lymphocyte count decreased	1 (16.7)	1 (16.7)	4 (33.3)	4 (33.3)	5 (27.8)	5 (27.8)
Neutrophil count decreased	6 (100.0)	6 (100.0)	11 (91.7)	9 (75.0)	17 (94.4)	15 (83.3)
Platelet count decreased	2 (33.3)	1 (16.7)	7 (58.3)	3 (25.0)	9 (50.0)	4 (22.2)
White blood cell count decreased	5 (83.3)	5 (83.3)	9 (75.0)	8 (66.7)	14 (77.8)	13 (72.2)
Metabolism and nutrition disorders						
Decreased appetite	6 (100.0)	1 (16.7)	7 (58.3)	0	13 (72.2)	1 (5.6)
Musculoskeletal and connective tissue disorders						
Myalgia	1 (16.7)	0	4 (33.3)	0	5 (27.8)	0
Nervous system and psychiatric disorders						
Headache	2 (33.3)	0	2 (16.7)	0	4 (22.2)	0
Psychiatric disorders						
Insomnia	2 (33.3)	0	3 (25.0)	0	5 (27.8)	0
Skin and subcutaneous tissue disorders						
Pigmentation disorder	2 (33.3)	0	0	0	2 (11.1)	0

All data are expressed as *n* (%). Adverse events were coded according to preferred terms in the Medical Dictionary for Drug Regulatory Activities (MedDRA), version 22.1

*ALT* alanine aminotransferase, *AST* aspartate aminotransferase, *CPK* creatine phosphokinase, *GGT* gamma-glutamyltransferase, *LDH* lactate dehydrogenase

Twenty serious AEs (SAEs) were reported in nine patients (two patients in level 1, and seven patients in level 2) (Supplementary Table S3). Treatment was discontinued due to SAEs in three patients (one patient with pseudoaldosteronism and one patient with arthritis bacterial in level 1, and one patient with female genital tract fistula in level 2). The other six patients with SAEs remained on treatment. No patients died during the trial.

### Pharmacokinetics

**Fig. 2:**
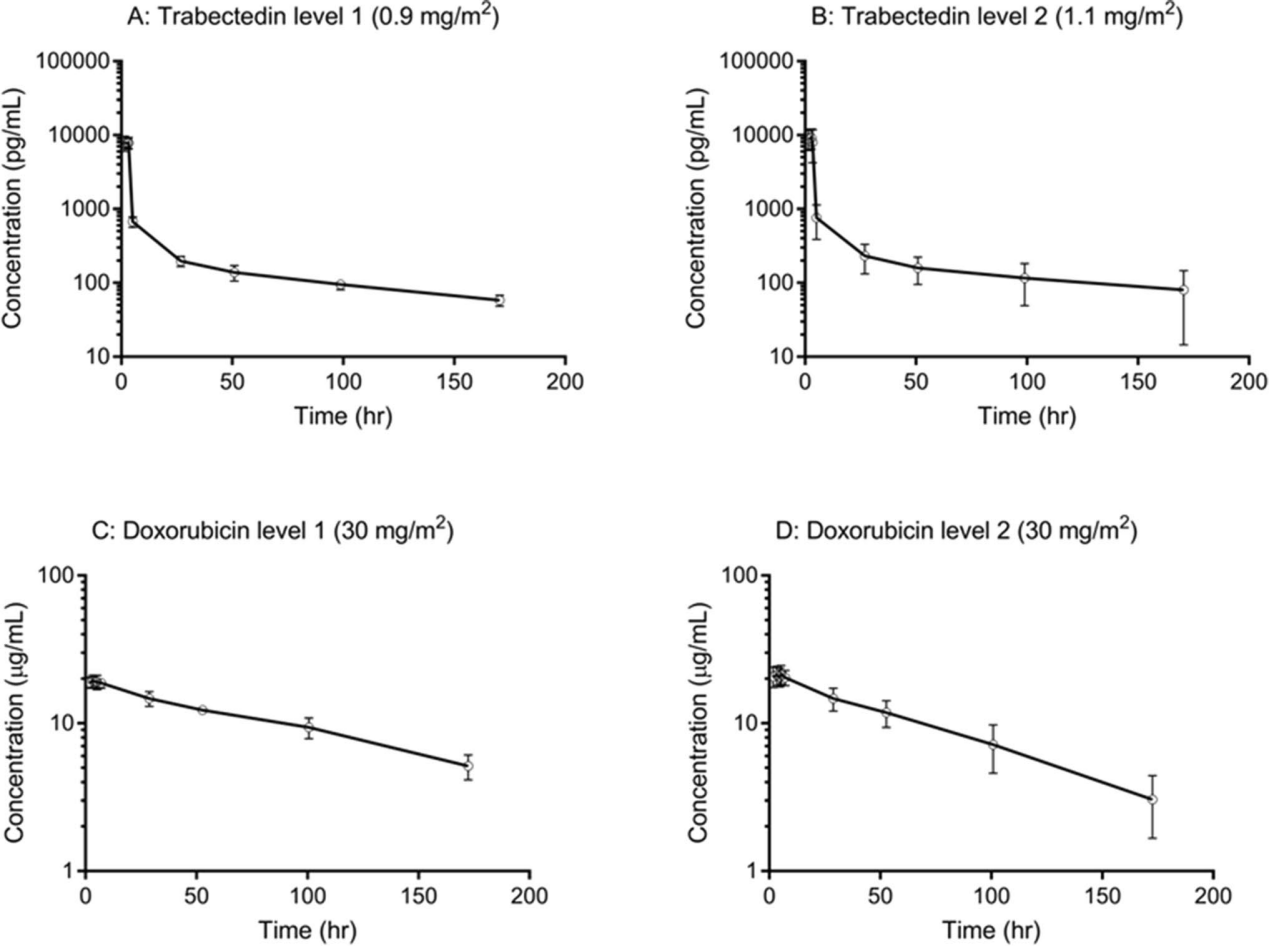
Mean plasma concentration–time profiles of trabectedin (panels **A** and **B**) and doxorubicin (panels **C** and **D**) in levels 1 (*n* = 6) and 2 (*n* = 12). Error bars indicate 1 standard deviation above and below the mean

Pharmacokinetic parameters are shown in Table 3. As illustrated in Fig. 2, trabectedin *C*_max_ was reached at the end of the 3-h infusion and declined rapidly thereafter; however, the mean *t*_½_ was > 100 h at both dosage levels, indicating gradual elimination.

**Table 3:** Pharmacokinetic parameters

Parameter	Trabectedin		Doxorubicin	
	Level 1 (*n* = 6)	Level 2 (*n* = 12)	Level 1 (*n* = 6)	Level 2 (*n* = 12)
*t*_max_, h	2.68 ± 0.94	2.78 ± 0.66	4.38 ± 1.70	4.37 ± 1.80
*C*_max_, *pg/mL or **µg/mL	8640 ± 1320*	9890 ± 3150*	19.8 ± 1.7**	22.2 ± 3.0**
AUC_0–inf_, *ng·h/mL or **µg·h/mL	61.5 ± 7.4*	83.7 ± 69.0*	2542.0 ± 391.2**	1941.2 ± 548.3**
*t*_½_, h	102.8 ± 27.8	124.9 ± 100.2	92.0 ± 15.3	61.0 ± 12.7
CL, *L/h or **mL/h	21.5 ± 2.2*	26.8 ± 9.3*	17.7 ± 3.4**	26.9 ± 9.1**
Vd_ss_, L	1461 ± 456	1921 ± 727	2.26 ± 0.27	2.15 ± 0.35

All data are expressed as mean ± SD

*AUC*_*0–inf*_ area under the concentration–time curve from zero to infinity, *CL* clearance, *C*_*max*_ maximum concentration, *SD* standard deviation, *t*_*½*_ terminal half-life, *t*_*max*_ time to *C*_max_, *Vd*_*ss*_ volume of distribution at steady state

Trabectedin exposure increased in a dose-dependent manner from 0.9 to 1.1 mg/m^2^. Inter-individual variation in both AUC_0-inf_ and *t*_½_ was greater in level 2 than level 1 because of a patient in level 2 with values (AUC_0-inf_ = 296.7 ng·h/mL and *t*_½_ = 431.3 h, respectively) that were ~ 3.5-fold higher than the mean.

Plasma concentrations of doxorubicin peaked ~ 4 h post-infusion and declined gradually (Fig. 2). Compared with published data [9, 10], pharmacokinetic parameters for doxorubicin were similar in level 2, but AUC_0–inf_ and *t*_½_ values in level 1 were higher (Table 3).

### Efficacy

**Fig. 3:**
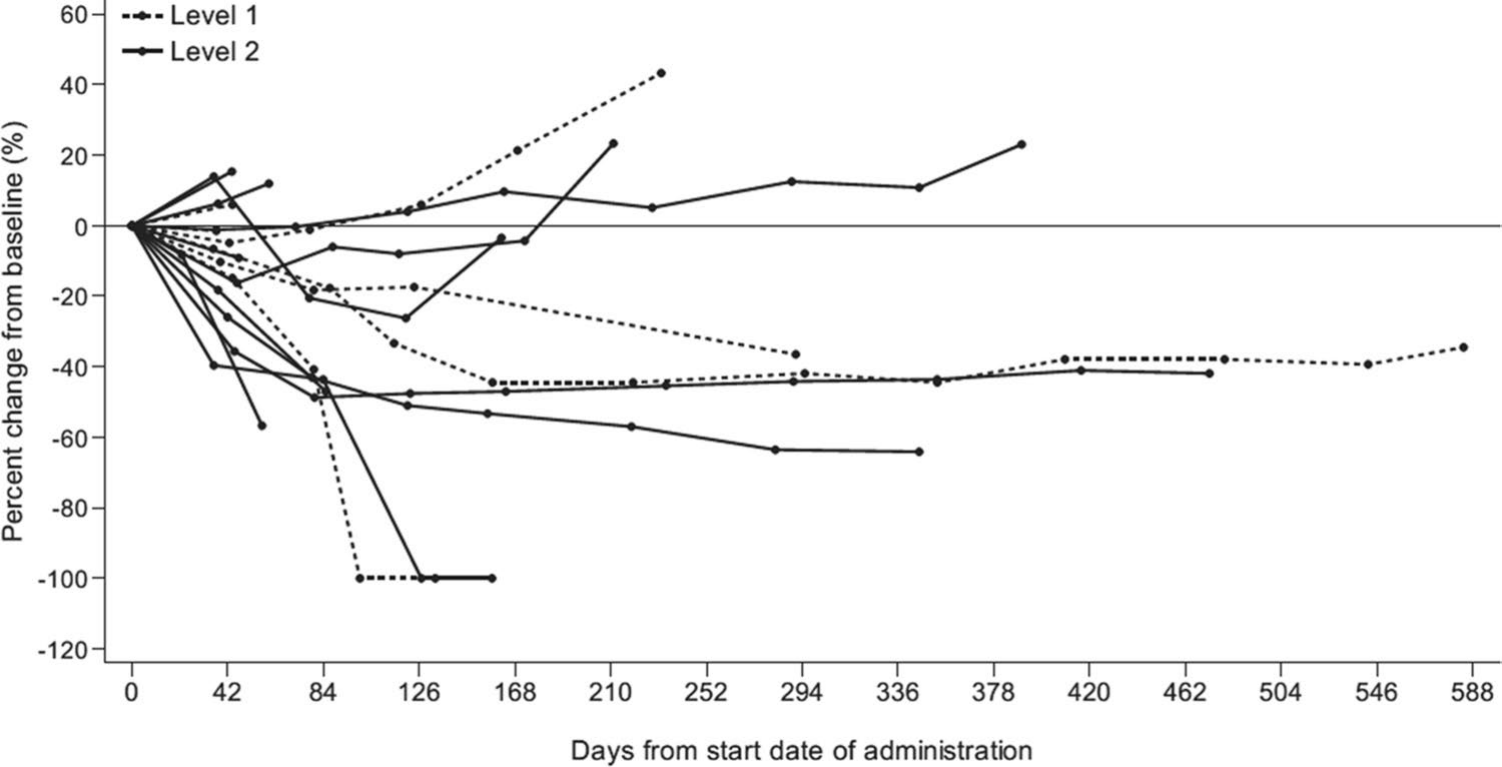
Percentage change from baseline in the sum of tumor diameters over time. Each line in the graph represents an individual patient; patients assigned to level 1 (trabectedin 0.9 mg/m^2^) are represented by dotted lines, and those assigned to level 2 trabectedin (1.1 mg/m^2^) are represented by solid lines. Data were missing for one patient (in level 2)

**Fig. 4:**
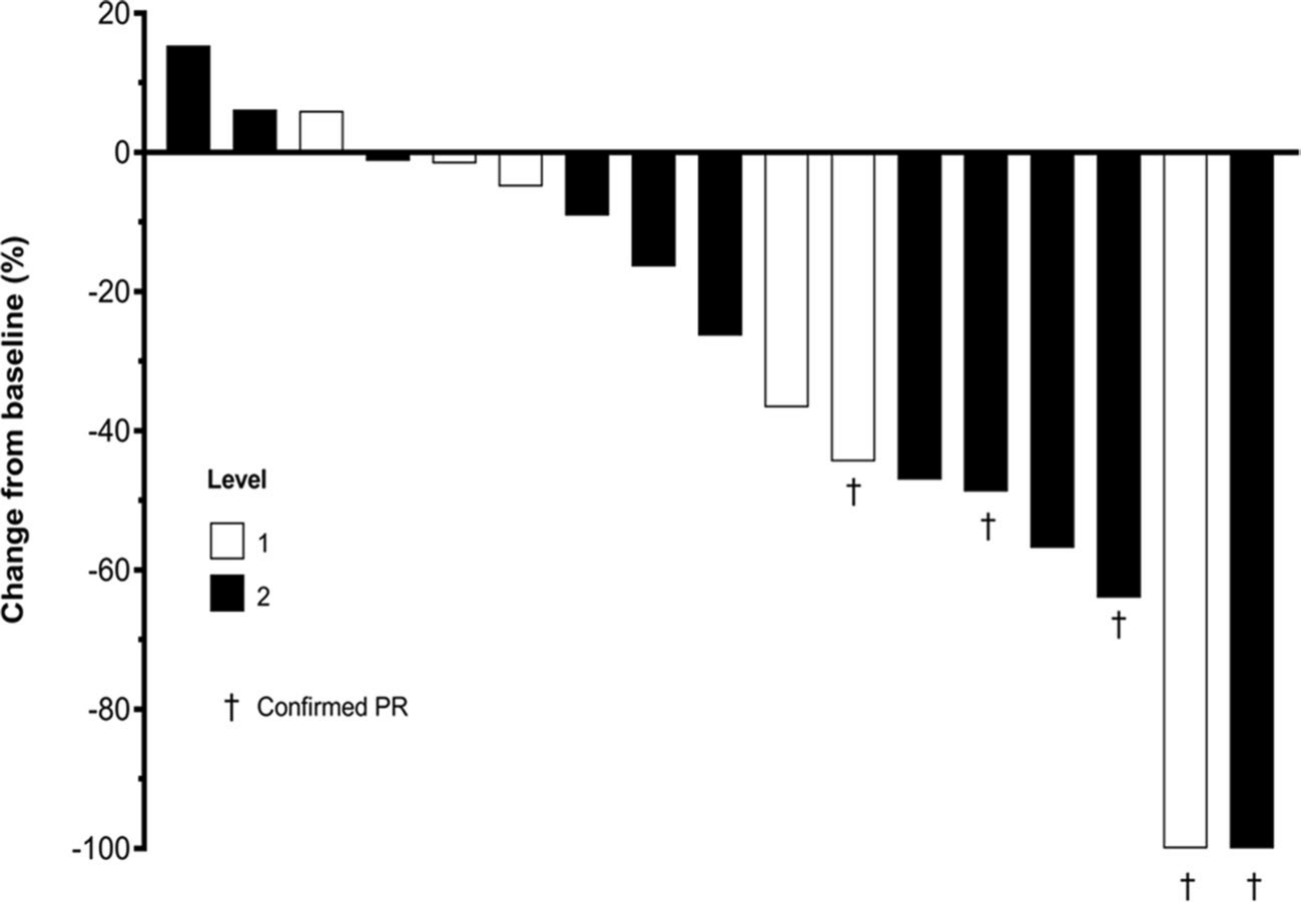
Best percentage change from baseline in the sum of tumor diameters. Each bar represents an individual patient. Data were missing for one patient (in level 2). *PR* partial response

Individual patient responses are shown in Figs. 3 and 4, and overall efficacy data are summarized in Table 4.

**Table 4:** Efficacy results

Outcome	Level 1 (*n* = 6)		Level 2 (*n* = 12)		Total (*n* = 18)	
	*n* (%)	95% CI (%)	*n* (%)	95% CI (%)	*n* (%)	95% CI (%)
Complete response (CR)	0	–	0	–	0	–
Partial response (PR)	2 (33.3)	–	3 (25.0)	–	5 (27.8)	–
Stable disease (SD)	2 (33.3)	–	6 (50.0)	–	8 (44.4)	–
Progressive disease	1 (16.7)	–	2 (16.7)	–	3 (16.7)	–
Not evaluable	1 (16.7)	–	1 (8.3)	–	2 (11.1)	–
Overall response rate (CR + PR)	2 (33.3)	4.3–77.7	3 (25.0)	5.5–57.2	5 (27.8)	9.7–53.5
Disease control rate (CR + PR + SD)	4 (66.7)	22.3–95.7	9 (75.0)	42.8–94.5	13 (72.2)	46.5–90.3

*CI* confidence interval

There were no complete responses. Five patients (two in level 1, and three in level 2) had a partial response (PR), giving an overall response rate (ORR) of 27.8% (33.3% [95% confidence interval (CI): 4.3–77.7%] in level 1 and 25.0% [95% CI: 5.5–57.2%] in level 2). In addition, eight patients (two in level 1, and six in level 2) had stable disease (SD), yielding an overall disease control rate (DCR) of 72.2% (66.7% [95% CI: 22.3–95.7%] in level 1 and 75.0% [95% CI: 42.8–94.5%] in level 2).

Four patients in level 2 had received prior treatment with PLD. All had a best overall response of SD, giving an ORR of 0.0% (95% CI: 0.0–60.2%). Conversely, eight patients in level 2 had not received PLD as prior medication; three patients had a PR, giving an ORR of 37.5% (95% CI: 8.5–75.5%).

Of the nine patients in level 2 with papillary/serous histology, three had a PR and five had SD, yielding an ORR of 33.3% and DCR of 88.9%. None of the three patients with clear cell carcinoma (*n* = 2) or mixed epithelial tumor (*n* = 1) responded to treatment.

## Discussion

No DLTs were observed with trabectedin 1.1 mg/m^2^ during the DEP; this dosage was therefore considered to be the MTD, and was used in the SAP.

Overall, no new safety concerns were identified, suggesting that trabectedin 1.1 mg/m^2^ plus PLD 30 mg/m^2^ is a viable treatment option for advanced relapsed OC in Japanese patients. Clinical AEs were either low grade (grade 1 or 2) and did not require intervention, or were more severe but resolved with appropriate management, including (if appropriate) reduction in the dosage of trabectedin and/or PLD. Malaise and fatigue were grade 1 or 2, while there were three grade 3 or 4 gastrointestinal AEs.

Most grade 3 or 4 AEs in our study were laboratory abnormalities. Comparison with previous trial findings [11, 12] suggests that combining trabectedin with PLD may cause more severe and/or more frequent cytopenias and other laboratory AEs compared with trabectedin or PLD alone. On balance, however, our safety findings were consistent with those of phase III trials of trabectedin with PLD in patients with relapsed OC [13, 14].

Nine patients experienced SAEs during the trial, but no patients died. Two cases of febrile neutropenia were considered serious: both were grade 3 events in patients who received trabectedin 1.1 mg/m^2^, and resolved with appropriate treatment (including antibiotics and granulocyte colony-stimulating factor). Neutropenia associated with trabectedin plus PLD, therefore, appears to be manageable in this population, even when severe.

Marked elevations in liver enzyme levels were frequent as in trabectedin monotherapy; all patients in level 2 and 50% of those in level 1 had grade 3 or 4 elevations in ALT. Encouragingly, however, there were no Hy's Law cases [15]. AST and ALT levels peaked at around day 3, but recovered rapidly (within 1–2 weeks) regardless of treatment. There were no cases of liver failure. Similarly, we found no evidence of severe rhabdomyolysis: two patients (both in level 2) had grade 3 elevations in creatine phosphokinase levels that were classed as serious, but both cases resolved completely and were not associated with clinical sequelae.

Trabectedin shows biphasic elimination, being rapidly redistributed from the plasma to the tissue compartment in the first phase and undergoing redistribution and gradual elimination in the second. The AUC_0-inf_ of doxorubicin was higher in level 1 compared with level 2, even though the PLD dosage was the same (30 mg/m^2^). Maximum doxorubicin concentrations were comparable between the two levels, but *t*_½_ was longer in level 1 (92 vs 61 h); thus, the observed difference in AUC_0–inf_ may be due to slower elimination in level 1. The published elimination *t*_½_ of doxorubicin, following the administration of PLD 30 mg/m^2^ to patients with solid tumors, is approximately 59 h [9], similar to the *t*_½_ in level 2. Patients in level 1 were, on average, older than in level 2 (mean age 63.0 vs 50.3 years, respectively), so differences in hepatic function may partly explain our observations. The unbalance of patient population between levels could be attributable to the small sample size.

Although the trial was not specifically designed or powered to assess efficacy, we nevertheless analyzed response rates based on the ‘best overall response’ method. The ORR was 25.0% in level 2, which is comparable with the ORR achieved in the OVA-301 trial (27.6%) [14] but lower than that in the OVC-3006 trial (46.0%) [13]; however, subjects in the latter trial were platinum-sensitive. These rates are higher than those reported with PLD monotherapy in either Japanese or non-Japanese patients with relapsed OC [12, 14].

Over 80% of the patients in our study were classed as platinum-resistant (i.e. PFI < 6 months). Our results suggest that trabectedin plus PLD may achieve higher ORRs in these patients than are typically achieved with currently recommended monotherapies (e.g. irinotecan, gemcitabine and topotecan) [16–18], but this needs to be addressed in an appropriately designed, adequately powered clinical trial. As mentioned earlier, trabectedin plus PLD has been approved in Europe for women with platinum-sensitive relapsed OC [5]; we have demonstrated that this combination is tolerable in Japanese patients, and further investigation of its efficacy in this population is warranted.

Based on an overall evaluation of the safety, tolerability, pharmacokinetic and efficacy data from this trial, we conclude that the RD of trabectedin, when used in combination with PLD in Japanese patients with relapsed OC, should be 1.1 mg/m^2^ by IV infusion every 21 days.

## Supplementary Information

Below is the link to the electronic supplementary material.

Supplementary file1 (DOCX 83 KB)

Supplementary file2 (DOCX 26 KB)

Supplementary file3 (DOCX 27 KB)

Supplementary file4 (DOCX 29 KB)
