# Supplementary material for: A phase I study of combined trabectedin and pegylated liposomal doxorubicin therapy for advanced relapsed ovarian cancer
Source: Int J Clin Oncol. 2021 Jun 30;26(10):1977–85. doi: 10.1007/s10147-021-01973-1 (PMC8449774; doi:10.1007/s10147-021-01973-1)

## Supplementary Figure S1. Progression of study. All patients received trabectedin in combination with pegylated liposomal doxorubicin. DLT, dose-limiting toxicity; MTD, maximum tolerated dose. *In accordance with the trial protocol, 6 patients were enrolled at level 2 in the dose escalation phase, because this was presumed to be the MTD.


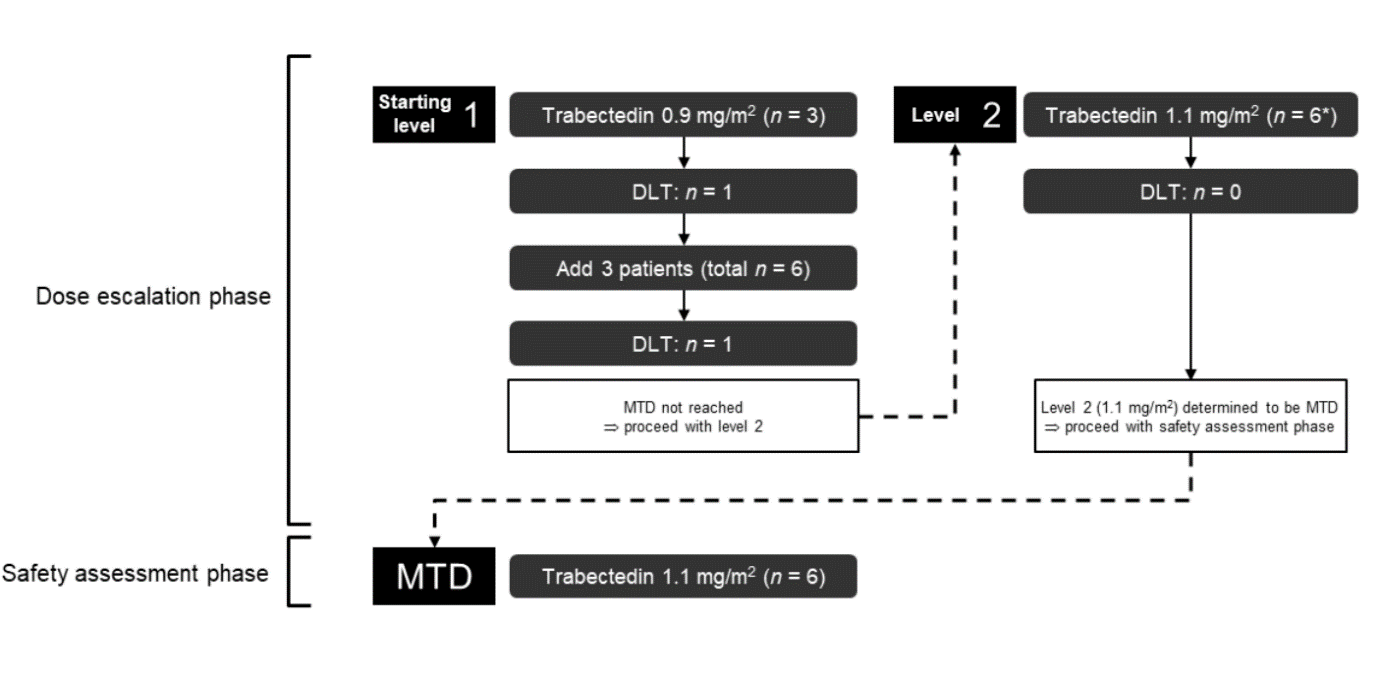

Supplement: Supplementary file 1 — Supplementary file1 (DOCX 83 KB) [file 10147_2021_1973_MOESM1_ESM.docx]
