# Supplementary material for: A phase I study of combined trabectedin and pegylated liposomal doxorubicin therapy for advanced relapsed ovarian cancer
Source: Int J Clin Oncol. 2021 Jun 30;26(10):1977–85. doi: 10.1007/s10147-021-01973-1 (PMC8449774; doi:10.1007/s10147-021-01973-1)
Supplement: Supplementary file 2 — Supplementary file2 (DOCX 26 KB) [file 10147_2021_1973_MOESM2_ESM.docx]

**Supplementary Table S1.** Definition of dose-limiting toxicities

| **Dose-limiting toxicities** | |
| --- | --- |
| **1** | Grade 4 decrease in neutrophil count persisting for at least 6 days |
| **2** | Grade 4 decrease in neutrophil count, accompanied by pyrexia ≥38.5°C or infection |
| **3** | Grade 4 decrease in platelet count, or grade 3 decrease in platelet count requiring transfusion |
| **4** | Grade ≥3 increase in aspartate aminotransferase (AST) or alanine aminotransferase (ALT) that did not improve to ≤2.5-fold of the upper limit of normal (ULN) by Day 28 |
| **5** | Other grade ≥3 non-haematological toxicity  **Note:** Grade 3 increases in alkaline phosphatase (ALP) or gamma-glutamyltransferase (GGT) and transient electrolyte abnormalities are excluded. Nausea, vomiting, diarrhoea and anorexia were considered to be dose-limiting if grade 3 toxicity persisted despite maximal supportive therapy |
