# Supplementary material for: A phase I study of combined trabectedin and pegylated liposomal doxorubicin therapy for advanced relapsed ovarian cancer
Source: Int J Clin Oncol. 2021 Jun 30;26(10):1977–85. doi: 10.1007/s10147-021-01973-1 (PMC8449774; doi:10.1007/s10147-021-01973-1)
Supplement: Supplementary file 3 — Supplementary file3 (DOCX 27 KB) [file 10147_2021_1973_MOESM3_ESM.docx]

**Supplementary Table S2.** Criteria for dosage reduction. Dosages of trabectedin were reduced one level at a time (i.e. from level 2 to level 1, or from level 1 to level -1), whereas dosages of pegylated liposomal doxorubicin (PLD) were reduced by 5 mg/m^2^ at a time (e.g. from 30 mg/m^2^ to 25 mg/m^2^)

| **Criteria:** |  |  | **Applies to:** | |
| --- | --- | --- | --- | --- |
| **Adverse event** | **Trigger for dose reduction** |  | **Trabectedin** | **PLD** |
| Platelet count decreased | Grade 4 |  | Yes | Yes |
| Neutrophil count decreased | Grade 4, lasting ≥6 days |  | Yes | Yes |
|  | Grade 4, with fever (≥38.5°C) or infection |  | Yes | Yes |
| ALT or AST increased | Grade ≥3 on Day 15, reduced to grade ≤1 by Day 43 |  | Yes | Yes |
| Total bilirubin increased | >1.5 mg/dL |  | Yes | No |
| Stomatitis | Grade ≥3 |  | No | Yes |
| Hand-foot syndrome | Grade ≥3 |  | No | Yes |
|  | Grade ≥1 if grade ≥3 in a previous cycle |  | No | Yes |
| Nausea, vomiting | Grade ≥3 and treatment with antiemetic therapy not possible |  | Yes | Yes |

ALT, alanine aminotransferase; AST, aspartate aminotransferase.
