# Supplementary material for: A phase I study of combined trabectedin and pegylated liposomal doxorubicin therapy for advanced relapsed ovarian cancer
Source: Int J Clin Oncol. 2021 Jun 30;26(10):1977–85. doi: 10.1007/s10147-021-01973-1 (PMC8449774; doi:10.1007/s10147-021-01973-1)
Supplement: Supplementary file 4 — Supplementary file4 (DOCX 29 KB) [file 10147_2021_1973_MOESM4_ESM.docx]

**Supplementary Table S3.** Serious adverse events. Patients in level 1 began treatment with trabectedin 0.9 mg/m^2^ per cycle, while those in level 2 began treatment with 1.1 mg/m^2^. All patients also received pegylated liposomal doxorubicin (starting dose 30 mg/m^2^)

| **Patient #** | **Level** | **Adverse event** | **Grade** | **Day of first onset** | **Causality†** | **Action** | **Outcome** |
| --- | --- | --- | --- | --- | --- | --- | --- |
| 1 | 1 | Decreased appetite | 3 | 3 | Reasonably possible | None | Recovered/resolved |
|  |  | AST increased | 4 | 3 | Reasonably possible | None | Recovered/resolved |
|  |  | ALT increased | 4 | 3 | Reasonably possible | None | Recovered/resolved |
|  |  | Pseudoaldosteronism | 3 | 37 | Not reasonably possible | Discontinued | Recovering/resolving |
| 2 | 1 | Arthritis bacterial | 3 | 281 | Reasonably possible | Discontinued | Recovering/resolving |
| 3 | 2 | Febrile neutropenia | 3 | 36 | Reasonably possible | None | Recovered/resolved |
|  |  | Platelet count decreased | 4 | 33 | Reasonably possible | None | Recovered/resolved |
|  |  | Blood CPK increased | 3 | 33 | Reasonably possible | None | Recovered/resolved |
| 4 | 2 | AST increased | 4 | 3 | Reasonably possible | None | Recovered/resolved |
|  |  | ALT increased | 4 | 3 | Reasonably possible | None | Recovered/resolved |
| 5 | 2 | Vomiting | 2 | 12 | Not reasonably possible | None | Recovering/resolving |
| 6 | 2 | Hypoxia | 2 | 59 | Not reasonably possible | None | Not recovered/not resolved |
| 7 | 2 | Gastrointestinal haemorrhage | 2 | 37 | Reasonably possible | None | Recovered/resolved |
|  |  | Decreased appetite | 2 | 64 | Reasonably possible | None | Recovered/resolved |
|  |  | Febrile neutropenia | 3 | 64 | Reasonably possible | None | Recovered/resolved |
|  |  | Nausea | 2 | 159 | Reasonably possible | None | Recovered/resolved |
|  |  | Female genital tract fistula | 2 | 173 | Not reasonably possible | Discontinued | Recovering/resolving |
| 8 | 2 | Blood CPK increased | 2 | 44 | Reasonably possible | None | Recovered/resolved |
| 9 | 2 | Rectal perforation | 3 | 387 | Not reasonably possible | None | Recovered/resolved |
|  |  | Hydronephrosis | 3 | 387 | Not reasonably possible | None | Recovered/resolved |

† With respect to trabectedin. Causality with respect to pegylated liposomal doxorubicin is not shown.

ALT, alanine aminotransferase; AST, aspartate aminotransferase; CPK, creatine phosphokinase.
